# Social Stories in mainstream schools for children with autism spectrum disorder: a feasibility randomised controlled trial

**DOI:** 10.1136/bmjopen-2016-011748

**Published:** 2016-08-11

**Authors:** David Marshall, Barry Wright, Victoria Allgar, Joy Adamson, Christine Williams, Hannah Ainsworth, Liz Cook, Danielle Varley, Lisa Hackney, Paul Dempster, Shehzad Ali, Dominic Trepel, Danielle Collingridge Moore, Elizabeth Littlewood, Dean McMillan

**Affiliations:** 1 Lime Trees CAMHS Research Team, York, UK; 2 Department of Health Sciences, University of York, York, UK

**Keywords:** autism spectrum disorder, feasibility randomised controlled trial, Social Stories, social competence, school based intervention

## Abstract

**Objectives:**

To assess the feasibility of recruitment, retention, outcome measures and intervention training/delivery among teachers, parents and children. To calculate a sample size estimation for full trial.

**Design:**

A single-centre, unblinded, cluster feasibility randomised controlled trial examining Social Stories delivered within a school environment compared with an attentional control.

**Setting:**

37 primary schools in York, UK.

**Participants:**

50 participants were recruited and a cluster randomisation approach by school was examined. Participants were randomised into the treatment group (n=23) or a waiting list control group (n=27).

**Outcome measures:**

Acceptability and feasibility of the trial, intervention and of measurements required to assess outcomes in a definitive trial.

**Results:**

An assessment of the questionnaire completion rates indicated teachers would be most appropriate to complete the primary outcome measure. 2 outcome measures: the Social Responsiveness Scale (SRS)-2 and a goal-based measure showed both the highest levels of completion rates (above 80%) at the primary follow-up point (6 weeks postintervention) and captured relevant social and behaviour outcomes. Power calculations were based on these 2 outcome measures leading to a total proposed sample size of 180 participant groups.

**Conclusions:**

Results suggest that a future trial would be feasible to conduct and could inform the policy and practice of using Social Stories in mainstream schools.

**Trial registration number:**

ISRCTN96286707; Results.

**Strengths and limitations of this study**

- The study used extensive qualitative evidence and patient and public involvement in conjunction with feasibility data when making conclusions.
- The study addressed an under-researched area and produced important feasibility evidence to inform the education of young people in the UK.
- The study provided valuable information to inform a future trial design and methods.
- The sample of participants was obtained from only one National Health Service (NHS) Trust resulting in potential for minorities to be under-represented.
- Blinding of participants to the intervention was not feasible due to the nature of the intervention.

## Introduction

Autism spectrum disorder (ASD) is a neurodevelopmental condition affecting 157 in every 10 000 children in the UK.^1^ These children are often characterised by difficulties in their social and emotional competence. Consequently, they are less able to intuitively understand societal norms as their typically developing peers.^2^ The current policy to assist social and academic development is to include them in mainstream classrooms,^3^ but there is growing evidence to suggest that without support such placements increase the risk of isolation and rejection.^4^

A Social Story is a non-intrusive, easy to implement intervention that appears to be effective for providing social information to children with ASD.^5^ A Social Story is a brief, individualised narrative that details a social situation and guides the child's behaviour through visual supports and text.^6^ They are commonly used in schools in the UK and have been recommended by a review of evidenced-based interventions.^7^ Their design uses 10 criteria to ensure that the story's structure and content is descriptive, meaningful, safe and constructive for those reading it.^8^ These criteria include rules for the type and balance of sentences throughout the text as well as specific language to avoid.

Until recently, research exploring the effectiveness of Social Stories has mainly been through use of single case design methodology. There is a paucity of good quality, comparative evidence of Social Stories particularly within school settings as highlighted in three systematic reviews of effectiveness.^5^ ^9^ ^10^ These reviews indicated an overall positive effect with individual case studies on a number of social and behavioural outcomes. Wright *et al*^10^ also reported on seven between-group studies, four of which were randomised controlled trials (RCTs).^11–14^ However, the literature is largely US based and the studies have generally failed to successfully follow Gray's criteria. In particular, interventions have tended to lack an individualised story constructed for the specific needs of the child and were vulnerable to selection and reporting bias.

From this evidence base and the recommendations of Kasari and Smith,^15^ there was a strong justification to conduct a well-designed, ecologically valid, large-scale RCT on the effectiveness of Social Stories which used individualised stories within a school setting. However, the individualised nature of Social Stories leads to complexities for their measurement and delivery. Therefore, there is a need to first conduct a feasibility RCT. Consequently, we designed the Autism Spectrum Social Stories in Schools Pilot Trial (ASSSIST) as a two-phase study to first adapt and develop the intervention for a UK population. In phase 2, we examined the feasibility of conducting a full scale trial. This paper focuses on the second phase of ASSSIST to assess the feasibility of delivering a RCT comparing the manualised Social Stories intervention with an attention control for children with ASD in mainstream schools. The feasibility objectives included recruitment, retention, outcome selection, sample size estimation for full trial and acceptability of intervention training and delivery among teachers, parents and children.^16^

## Methods

### Feasibility trial design

The feasibility study was a single-centre, unblinded, cluster RCT, comparing the feasibility of measuring the effectiveness and cost-effectiveness of Social Stories at altering the behaviour of children with ASD in a mainstream school setting with a comparator group using an attention control. The study had a nested qualitative study to examine acceptability. The study was conducted between November 2011 and October 2014.

### Participants

For each participating child, there was an associated teacher and parent who completed questionnaires and delivered the intervention. For ease of reference, these are called participant groups (one child, one parent and one teacher). We use the term teacher to refer to any school staff member designated to work with the participating child on the study (encompassing teaching assistants and special educational needs coordinators, as well as class teachers).

#### Inclusion criteria

Participant groups were included if the child: (1) had a diagnosis of ASD given by the multidisciplinary, multiagency York Autism Spectrum Disorders Forum or other equivalent bodies using the International Classification of Diseases (ICD) 10,^17^ or the Diagnostic and Statistical Manual of Mental Disorders (DSM)-IV,^18^ research diagnostic criteria, (2) was aged between 5 and 15 years, (3) attended mainstream school and (4) exhibited social interaction difficulties that resulted in problems at school as reported by parents or teachers.

#### Exclusion criteria

Participant groups were excluded if: (1) the child had used Social Stories in the preceding 6 months, (2) the child was likely to move schools in the following 4 months and (3) either the parent or teacher of the child had taken part in the qualitative interviews or focus groups conducted in the first phase of ASSSIST.

### Sample size

We aimed to recruit a total of 50 participant groups. This sample size was chosen based on the recommendations for feasibility studies by Sim and Lewis.^19^

### Setting

The participating teachers delivered the Social Stories within the mainstream classrooms. Professional educational and Child and Adolescent Mental Health Service (CAMHS) clinical staff associated with the research team delivered the training in Social Stories in a CAMHS setting.

### Qualitative component

To examine the acceptability of the intervention and the trial process, we purposively sampled parents (or carers) and their children in receipt of intervention (or comparator) from participants in the feasibility RCT (n=10). This sample included parents who were actively involved in supporting the intervention at home. We recruited five parents/children from the control arm as a comparator to examine the trial processes. We purposively sampled from the group of professionals who were delivering the intervention (n=5) to achieve a rounded picture of the intervention delivery from all key stakeholders across a series of cases from the study.

### Participant recruitment

Three alternative recruitment procedures were used to identify and recruit participants: contact through schools, contact through parent groups, and referrals from clinicians and local authority staff.

### Feasibility trial process

#### Goal setting meeting

A goal setting meeting (GSM) was arranged for all eligible participants to set a unique goal for the child to achieve by consensus between the teacher and the parent, and second, to define and operationalise specific behaviours that they hoped to increase and decrease. The GSMs lasted ∼90 min.

#### Randomisation and allocation concealment

To minimise contamination bias, cluster randomisation by school was adopted. Following completion of baseline measures, school randomisation using minimisation was used to account for the numbers of children with ASD, levels of support, the socioeconomic position of the area in which the school was situated (as measured by the deprivation indices) and the school's academic achievement (as measured by their value added measures). Allocation to groups was conducted remotely by York Trials Unit.

#### Intervention

An individualised Social Story was created for each child in the intervention arm (23 in total) to achieve an identified goal. The stories were created by the participating teachers with support from parents and researchers during a training day. All participating teachers left this training day with a fully constructed narrative for the Social Story that had been examined and validated by an expert trainer. Guidance was provided on how to format the story to individualise it to the particular child's needs (eg, story book format, power point slides or on a single sheet of A4 paper). All adult participants in the intervention arm also received a copy of the Social Stories training manual developed in phase 1 of ASSSIST.

The delivery of the intervention within schools was conducted by the participating teacher in accordance with their knowledge of the child's needs and abilities with support and advice from parents. The guidelines given to participating teachers were to read the story with the child three times a week at school for 2 weeks.

#### Comparator group

Participating teachers in the comparator group were asked to choose a typical age appropriate story of interest to the child (without any social information relevant to the goal) and were given similar guidance on reading it to those in the intervention group. Restrictions were not imposed on the length of this story but the teacher was advised to spend between 5 and 10 min of time reading with the child. A letter detailing how to present this story was sent to the teachers and parents and this included a date to start delivering the attention control.

### Outcome measure selection

Baseline questionnaires were given to participants in the GSM to be completed either at the end of the session or at home. Follow-up questionnaires (including all baselines measures) were sent to all participants at 6 and 16 weeks following the start of the intervention (or control). Participating children of all ability levels were asked to complete some measures to assess the feasibility of collecting data from this population in a larger trial.

#### Potential primary outcome measures

The following measures were considered as potentially appropriate to be selected as the primary outcome in a full scale RCT and were therefore administered as part of the feasibility study:

1. The Strengths and Difficulties Questionnaire (SDQ):^20^ The SDQ is a 25-item measure of internalising and externalising difficulties, which includes five subscales. Four of these (emotional symptoms, conduct problems, hyperactivity/inattention and peer problems) were combined to obtain a total difficulties score ranging from 0 to 40. The mean and SD of this composite score was used in the analysis. It has strong reliability and validity as a dimensional measure of psychological constructs.^21^ The scale was completed by participating parents, teachers and older children (11–15 years).
2. The Social Responsiveness Scale-2 (SRS-2):^22^ The SRS-2 identifies social impairment associated with ASD and quantifies its severity. It is a 65-item scale and each item has four response options giving a score of 0–3. SRS total raw scores range from 0 to 195 and higher scores indicate increased social impairment. It has been included as a measure of ASD symptom severity and has been shown to have high reliability and validity for a UK population.^23^ It was completed by the teachers.
3. A goal-based outcome measure, designed by the research team, to enable individualised, prespecified goals to be recorded and measured on an 11-point Likert scale (0–10). The goals were specified in a GSM prior to entry into the trial. A score of 10 indicated the goal was met and 0 indicated it was not at all met. This was included as a measure of perceived effect of the intervention. It was completed by all participants including the children.
4. A behavioural frequency measure (BFM), designed by the research team, to enable individualised goals and measured on five-point Likert scales. The points on the scales gave an indication as to the frequency of the behaviour from ‘never’ to ‘very frequently’. There were two of these scales: one of which measured a desired behaviour and one which assessed a challenging behaviour. These were included as a measure of perceived effect of the intervention. Both were completed by the adult participants.
5. A diary, designed by the research team, to collect data over the course of a week on the frequency of delivery of the stories and the frequency of occurrence of prespecified behaviours across the school day. The measure was included to examine the logistical feasibility of an observation-based measure in a full trial. It was completed by the adult participants.

#### Economic outcome measures

1. The EQ-5D proxy and the EQ-5DY:^24^ These are standardised five-item instruments for use as measures of generic health outcomes recommended by the National Institute for Health and Care Excellence (NICE). Higher scores indicate more health problems. They were completed by the participating parents (EQ-5D proxy) and children (EQ-5DY), respectively.
2. Health Utilities Index 2 (HUI2):^25^ This is a preference-based generic health outcome measure to establish health states in children, report health-related quality of life and related produce utility scores. Higher scores indicate more health problems. It was completed by the participating parents.
3. Bespoke resource use questionnaires were developed by the research team's health economist to capture the resource implications of the child's behavioural problems at school and home. These questionnaires were completed by the adult participants.

### Blinding

Blinding of the participants (inclusive of teachers, parents and children) was not feasible due to the nature of the intervention. Members of the study team responsible for the statistical and economic analysis were kept blind to group allocation.

### Statistical analysis

#### Clinical outcomes analysis

We reported the flow of participants through the study according to the Consolidated Standards of Reporting Trials (CONSORT) guidelines for non-pharmaceutical interventions. In line with recommendations about good practice in the analysis of feasibility studies,^26^ no statistical comparisons of the outcomes between the two arms of the trial were conducted. Descriptive statistics were calculated for recruitment rates, follow-up rates, attrition and for baseline characteristics. These are presented as means and SDs for continuous data or number and percentage for categorical data. Descriptive statistics are also calculated for the outcome measures. All analyses were undertaken on SPSS (V.22).

#### Economics analysis

The feasibility of conducting an economic analysis was examined alongside this feasibility RCT to inform the choice of appropriate measures of generic health and identify the relevant resource use categories for a full trial cost-effectiveness analysis. The feasibility and challenges of measuring costs and outcomes in the target population was also examined.

#### Qualitative analysis

Interviews were audio-recorded and fully transcribed. Transcripts were read and key points were coded according to a priori themes based on the aims of the process evaluation using NVivo V.10,^27^ using a framework approach with two coders.^10^ ^28^ Coding related to the acceptability of the training for and delivery of intervention among parents, teachers and children where appropriate, and factors relating to the conduct of the trial including recruitment, information provided, randomisation and outcome measurement.

### Adverse events

Adult participants were provided with a contact number to ring if they or the child had any concerns about the trial. These were conducted with teachers, parents and where applicable the children. Further analysis of the diaries was also conducted. Any concerns raised were dealt with either by members of the research team or referred to an appropriate clinician or services as per usual practice. Details of all adverse events were recorded.

## Results

### Participant recruitment

**Figure 1:**
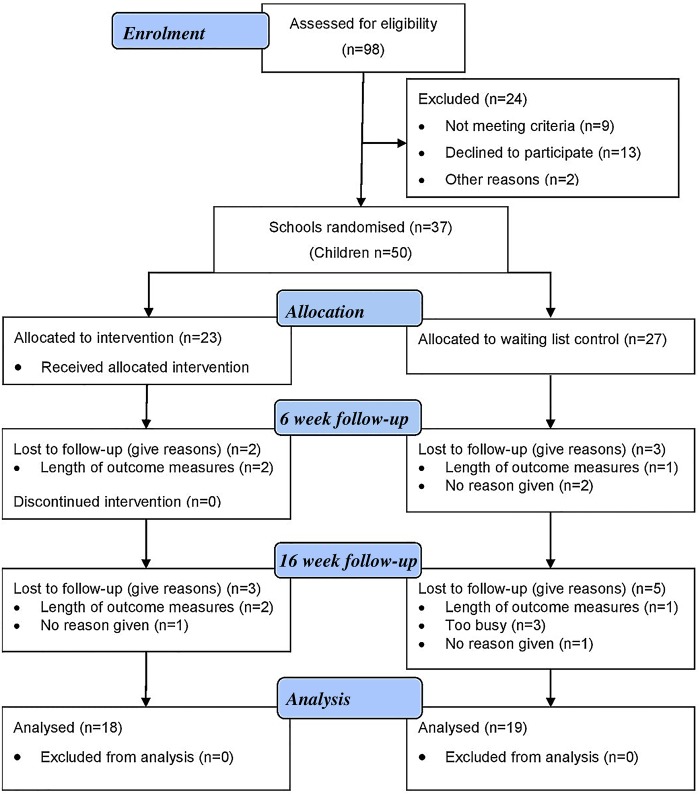
CONSORT flow diagram.

In total, 140 potentially eligible participant groups were identified and contacted. Of these, 66 did not respond to contact and could not be assessed for eligibility. A full breakdown of recruitment is summarised in figure 1. Thirty-three of these participant groups were recruited through direct contact with schools, 10 through parent groups and 8 through referrals by educational and clinical specialists.

### Baseline characteristics

#### Characteristics of clusters

Clusters ranged in size from one to four participant groups with a mean of 1.35 children with ASD per cluster. Twenty-five of the recruited clusters were primary schools, nine were secondary schools and three were private/independent schools.

#### Characteristics of participants

Table 1 summarises the characteristics of the child participants recruited to the trial. The majority of these participants were male (74%) which is consistent with the distribution of ASD in the population.^29^ Most of the participants were recruited from primary schools (62%). The participants assigned to the comparator group were slightly older (M=9.9) than the intervention group (M=9.2).

**Table 1:** Characteristics of the participants recruited to the feasibility trial

	Intervention group	Comparator group	Total
Gender			
Male	19 (83%)	18 (67%)	37 (74%)
Female	4 (17%)	9 (33%)	13 (26%)
Age at study entry (years)			
Mean	9.2 (2.7)	9.9 (2.5)	9.5 (2.6)
School type			
Primary schools	16 (70%)	15 (56%)	31 (62%)
Secondary schools	6 (26%)	10 (37%)	16 (32%)
Private/independent schools	1 (4%)	2 (7%)	3 (6%)

### Trial process

#### Participant flow and cluster design

There were some delays in the flow of participants from expressing interest in the trial through full enrolment to being allocated to a group. Owing to delays in baseline measure retrieval, it took between 6 and 14 weeks to be assigned a group and another 2–4 weeks after that for those in the intervention group to be trained.

All participants consented to randomisation and the resulting groups were equivalent in terms of their demographics. The requirement for completing all baseline questionnaires in each cluster before randomisation resulted in delays in treatment delivery for some participants and potentially to eligible participants being missed due to the limited timeframe. Additionally, the low average cluster size found across the recruited schools indicates there would be a relatively low impact of dilution bias on the results. As such, cluster randomisation by school was not considered necessary for a future trial.

#### Training, delivery and acceptability

Training sessions were well received by participants. All participant groups left the training session with a completed narrative for their story. No difficulties with implementing the intervention were reported to the researchers by participating teachers.

Because when we sat down to write it, between us, with the people being there to help, that, I think that was vital that they were there to help for that first sentence, that first story, but with their help I think we did OK ….

Female, teaching assistant, secondary school, intervention group

The overall perception was that the manual was useful in providing ancillary information, which would be particularly useful in the longer term, acting as an aide memoir for use beyond the training sessions, rather than use as a stand-alone item:

Yeah, it was clear and came away with a booklet, lots of examples in it, you know, so if, if, we did the majority of it in the session which I think is really crucial.

Female, parent, primary school, intervention group

To further test the feasibility of rolling out the study to a full scale trial, we examined the feasibility of training educational professionals in Social Stories training. A specialist autism teacher who had previously been involved in the user focus group and an educational psychologist were trained to deliver the training sessions by the clinicians on the research team. All of these sessions were monitored by the trial coordinator and had high fidelity to the training and learning objectives as measured by a predesigned fidelity.

#### Fidelity of the intervention

The adult participants constructed and revised their stories with support from the specialist trainers and other members of the research team, during the training session, to ensure that the finished product followed all of Gray's criteria.^8^ To examine drift from the criteria during the delivery of 20% of the stories in the intervention group, 22% of the stories were examined after they had been delivered. All remained consistent to Gray's criteria.^8^

#### Goal setting

Goal setting was seen as useful in that it encouraged people to meet around the table and ‘bounce’ ideas off each other. The process of goal setting itself was difficult due to a requirement for a goal applicable to school and home, especially when all participants could not attend:

I said, [to the researchers] ‘Can we tweak it a bit because {the child is] never going to get a piece finished if he doesn't get started?’ because that was my issue. I mean if I had been at the meeting I maybe could have put that point forward.

Female, teaching assistant, primary school, intervention group

#### Retention of participants

Questionnaire return rates are displayed in table 2 and were similar across the two groups at each time point. With regard to baseline measurements, all 50 (100%) participating teachers returned the questionnaires. A slightly lower response rate was seen from participating parents at baseline and the participating children had the lowest response rate.

**Table 2:** Summary of questionnaire and diary return rates

		Baseline			6 weeks			16 weeks		
Participant type	Measure	Intervention (%)	Comparator (%)	Total (%)	Intervention (%)	Comparator (%)	Total (%)	Intervention (%)	Comparator (%)	Total (%)
Teacher	Booklet	23 (100)	27 (100)	50 (100)	21 (91)	24 (89)	45 (90)	18 (78)	19 (70)	37 (74)
	Diary	13/19 (68)	12/19 (63)	25/38 (66)	15/19 (79)	13/19 (68)	28/38 (74)	8/19 (42)	6/19 (32)	14/38 (37)
Parent	Booklet	21 (91)	25 (93)	46 (92)	16 (70)	21 (78)	37 (74)	14 (61)	16 (59)	30 (60)
	Diary	11/19 (58)	11/19 (58)	22/38 (58)	7/19 (37)	8/19 (42)	15/38 (40)	5/19 (26)	4/19 (21)	9/38 (24)
Child	Booklet	18 (78)	18 (67)	36 (72)	12 (52)	15 (55)	27 (54)	7 (30)	10 (37)	17 (34)

With regard to baseline behaviour diaries, at the 16-week follow-up point, the return rates were low, particularly among parents (n=9 (24%)) of the parents returned them.

Teachers remarked the diaries were not found to be user friendly and difficult to complete. From some of the interviews, it was clear that the paperwork did not fit the specified behaviour goals and observations were difficult:

It was all about winning and losing and with, with him, we thought, I kinda set up a little thing, so we were looking at, you know, positive comments and negative comments and things and, you know, whether he was, whether he was winning and things, and also we had to, we had to set up some games which we wouldn't normally have kind of played.

Female, teaching assistant, primary school, intervention group

Therefore, the diaries were removed for the last 12 participant groups to test if this improved trial logistics.

#### Intervention delivery

For those randomised to the Social Story group, finding the time for reading the Social Story was important. It was seen as important to create ‘protected time’. This was more straightforward in a primary setting:

Yeah, it was, you know, obviously these children, sometimes they're in the right frame of mind and sometimes they're not. So I tried to do it in a roundabout way, just let's go and read the story, and made, just made it really exciting. So that helped. I think if, if I'd have said “Right, come on, we need to do the story before we do this task” then I think she would have…Twigged and thought no.

Female, teaching assistant, primary school

Within the secondary school, there was the added complication of finding an appropriate time and place to deliver the intervention.

Pupils at secondary school have a structured timetable there is little to no time to sit down with a pupil and really go through a social story. Pupils don’t tend to want to go through the stories in their break times and we feel that we can’t force them.

Female, teaching assistant, secondary school

#### Acceptability and perceived impact

For the children who had participated in the reading of Social Stories, most of the teachers/parents felt the stories were acceptable and had indeed achieved a positive outcome:

You could see the difference at the beginning of the week, at the end of the week.

Female, teaching assistant, primary school, intervention group

However, while it was not necessarily possible to attribute any change to the story per se, the intervention was not associated with any negative impacts.

### Primary outcome selection

#### Completion rates

In addition to retention rates, the completion rates of individual questionnaires were examined. Table 3 displays the individual breakdown of these figures. Only the goal-based outcome for participating teachers had overall completion rates ≥70% at each time point with the percentage completion rates between the intervention and comparator groups showing similar rates: 96–100% at baseline, 83–89% at 6 weeks and 70% at 16 weeks. There was a notable difference in the completion rates between the time points with follow-up points being much lower. There was also a notable difference in the completion rates for the SRS-2 (74% vs 63%) and BFM (74% and 67%) between the groups at 16-week follow-up. The participating parents had good completion rates at baseline on the potential primary outcome measures but less so at 6 and 16 weeks. The participating children had poor completion rates at all time points and some parents indicated that the validity of the data was questionable.

**Table 3:** Return and completion rates of individual questionnaires

		Baseline			6 weeks			16 weeks		
Participant type	Measure	Intervention (%)	Comparator (%)	Total (%)	Intervention (%)	Comparator (%)	Total (%)	Intervention (%)	Comparator (%)	Total (%)
Teacher	Goal-based	22 (96)	27 (100)	49 (98)	19 (83)	24 (89)	43 (86)	16 (70)	19 (70)	35 (70)
	SDQ	23 (100)	26 (96)	49 (98)	18 (78)	17 (63)	35 (70)	16 (70)	12 (44)	28 (56)
	SRS-2	23 (100)	27 (100)	50 (100)	21 (91)	20 (74)	41 (82)	17 (74)	17 (63)	34 (68)
	BFM	22 (96)	26 (96)	48 (96)	20 (87)	23 (85)	43 (86)	17 (74)	18 (67)	35 (70)
Parent	Goal-based	20 (87)	25 (93)	45 (90)	15 (65)	19 (70)	34 (68)	12 (52)	14 (52)	26 (52)
	SDQ	21 (91)	25 (93)	46 (92)	15 (65)	17 (63)	32 (64)	14 (61)	16 (59)	30 (60)
	BFM	20 (87)	24 (89)	44 (88)	14 (61)	19 (70)	33 (66)	12 (52)	14 (52)	26 (52)
Child	Goal-based	14 (61)	14 (52)	28 (56)	10 (43)	12 (44)	22 (44)	5 (22)	6 (22)	11 (22)
	SDQ	4 (17)	9 (33)	13 (26)	3 (13)	5 (19)	8 (16)	2 (9)	7 (16)	9 (18)

BFM, behavioural frequency measure; SDQ, Strengths and Difficulties Questionnaire; SRS, Social Responsiveness Scale.

While some people found the paperwork relating to outcome straightforward to fill out, most reported that they felt too much data were being collected. Many were willing to complete the questionnaires because of the perceived benefits to the research, but there were common symptoms about length, clarity and repetitive questions:

It's good because you need to know the outcome of the story but I think it's just too long.

Female, teaching assistant, primary school, intervention group

#### Descriptive statistics

Table 4 displays the mean and SD for each outcome measure across all time points. The teacher goal-based, SRS-2 and BFMs were at a high enough rate to be considered feasible for a full scale trial. The goal-based measure and SRS-2 showed change scores in the expected direction but the summary data indicate that the BFMs may not have been sensitive enough to detect change.

**Table 4:** Mean and SD of potential primary outcome measures at each time point

		Baseline		6 weeks		16 weeks	
Participant type	Measure	Intervention	Comparator	Intervention	Comparator	Intervention	Comparator
Teacher	Goal-based	1.9 (2.0)	2.8 (2.0)	6.5 (2.9)	5.4 (2.7)	7.3 (2.7)	5.4 (2.8)
	SDQ	2.6 (1.5)	2.5 (1.5)	0.6 (0.9)	0.5 (0.9)	0.2 (0.5)	0.8 (1.2)
	SRS-2	74.8 (9.6)	72.2 (10.7)	71.5 (9)	73.8 (10.1)	70.1 (10.7)	72.3 (10.9)
	BFM-DB	3.0 (1.33)	2.7 (1.02)	3.1 (0.92)	3.2 (0.93)	3.2 (0.95)	3.3 (0.87)
	BFM-CB	4.0 (1.11	3.5 (0.76)	3.0 (1.0)	3.0 (0.98)	3.0 (1.0)	3.1 (0.96)
Parent	Goal-based	2.1 (1.7)	2.5 (2.1)	5.9 (2.4)	4.3 (2.9)	7.2 (2.7)	4.0 (2.6)
	SDQ	4.9 (2.6)	4.4 (2.7)	0.9 (1.5)	0.2 (0.4)	0.7 (1.5)	0.5 (1.1)
	BFM-DB	2.8 (0.98)	3.1 (1.12)	3.4 (0.73)	2.8 (1.12)	3.5 (0.97)	2.8 (0.94)
	BFM-CB	3.6 (1.27)	3.9 (0.85)	3.2 (0.8)	3.6 (0.96)	3.3 (1.07)	3.3 (1.07)
Child	Goal-based	3.0 (2.8)	3.9 (2.4)	7.9 (3)	4.6 (2.8)	7.6 (2.1)	5.3 (2.3)
	SDQ	6.0 (2.9)	2.4 (2.4)	0.7 (1.2)	0.2 (0.4)	0.0 (0)	0.1 (0.4)

BFM, behavioural frequency measure; SDQ, Strengths and Difficulties Questionnaire; SRS, Social Responsiveness Scale.

#### Economic feasibility

In line with the overall rates of completion, the completion rate of the EQ-5D proxy at 6 weeks showed a total of 37 out of 50 (74%) and at 16 weeks, 30 out of 50 (60%). The completion rate of the HUI2^25^ was lower than the EQ-5D proxy. The total completion rate of the HUI2 was 32 (64%) at the 6-week follow-up, and 29 (58%) at the 16-week follow-up. In contrast to these measures but consistent with other child measures, the completion rate of the EQ-5DY was very low. At 6 weeks, 24 out of 50 (48%) children completed the questionnaire and at 16 weeks, 15 out of 50 (30%) completed them.

The ability to assess resource implications was piloted using a bespoke resource use questionnaire for parents and teachers. Examining the items relating to health services use at baseline, 6 and 16 weeks showed a high proportion of missing responses. Owing to the construction of the questionnaire, it was not possible to assess whether missing values represented no service usage. The feasibility of collecting resource-related information from participating teachers was promising to inform an extended perspective as they provided a good indication of school grades, the level of pupil productivity and how often participating children did not attend school.

### Power calculation for full scale trial

Calculations were undertaken based on the SRS T-score total score using the change in score from baseline to 6 weeks. We chose this measure to base our calculations on as the subjective nature of the goal-based measure makes it more difficult to state that an observable change is clinically significant. Table 5 showed the descriptive summary of the SRS total scores. The intervention group's scores changed by 5.28 (7.17) and by 1.95 (6.79) in the comparator group from baseline to 6 weeks. Using the difference in the change in scores of 3.33 (5.28–1.95), a sample size of 72 in each group would be required at 80% power to detect a difference of 3.33, assuming that the common SD is 6.975 using a two-group t-test with a 5% two-sided significance level. Allowing for 80% response rate, we require 90 participant groups=total sample size of 180. It was deemed inappropriate to continue with a cluster design and inflate this total further.

**Table 5:** Total SRS score for paired data at baseline and 6 weeks

	Group	N	Mean	SD
Baseline	Comparator	22	75.59	8.75
	Intervention	20	77.30	7.53
6 weeks	Comparator	19	74.37	10.01
	Intervention	18	72.28	8.84
Difference (6 weeks to baseline)	Comparator	19	−1.95	6.79
	Intervention	18	−5.28	7.17

SRS, Social Responsiveness Scale.

## Discussion

### Summary of findings

The purpose of this study was to test the feasibility of conducting a RCT examining the clinical and cost-effectiveness of Social Stories compared with an attentional control for children with ASD attending mainstream schools. To accomplish this, we examined methods of recruitment; the randomisation process; trial logistics; participant retention and completion of measures; and qualitative feedback. The results were generally positive but the process indicated substantial areas which needed improvement to scale up to a fully powered trial.

We tested three methods of recruitment and all three showed a degree of success. We were able to recruit to a target of 50 participant groups within the specified timeframe of 12 months. Recruitment was most effective when contacting schools directly. However, other strategies such as recruitment through parent support groups and professional referrals further augmented this process.

All participating teachers and the majority of parents attended a training session, completed a Social Story and reported that they successfully delivered the intervention within the intervention period. We were able to improve the trial logistics and speed up the process of participation through the removal of an outcome measure that required more time to administer, explain and complete than might be feasible for a full scale trial.

Retention of participant groups was good, with only one participant group withdrawing before randomisation. Questionnaire completion rates were highest for participating teachers, and good for participating parents. This is consistent with the findings of previous studies in the area.^30^ ^31^ However, the feasibility of collecting questionnaires from child participants was questionable due to low response rates and parent reports of low validity. Two outcome measures, the SRS-2^22^ and the custom-made goal-based measure, showed high levels of completion rates (for teachers), good face validity and a trend in the desired direction indicating that they would be suitable for use as primary outcome measures in a full scale trial. Power calculations were conducted using the SRS-2 to give a total required sample size of 180 participants for a full scale trial.

### Limitations of the feasibility study

There was a substantial delay in treatment for some participants. This delay was attributed to a combination of reasons such as the difficulty in arranging GSMs, teacher availability for training, the requirement for diary completion and the cluster design. This was substantially improved by the removal of the diary measure and could be improved further by using simple randomisation in a full scale trial.

Intervention fidelity with regard to if the stories remained consistent to the criteria throughout the delivery was only partially assessed. It would be important to include a time point for all stories to be checked again after delivery if a full scale trial were to be conducted.

We did not collect data on the child's ability levels and this might be considered a limitation. We chose not to do this as we wanted to use a pragmatic, inclusive approach to explore if collecting data from this population of children was feasible. Consequently, it was deemed unnecessary to increase participant burden by the inclusion of these measures.

A further limitation was that this sample was obtained from only one NHS Trust resulting in potential for minorities to be under-represented. This would need to be addressed in a future full scale trial by expanding the study to a national level through use of multiple sites.

## Conclusions

Despite the limitations, this study is the first to systematically evaluate the feasibility and acceptability for examining the effects of Social Stories in a school environment. To the best of our knowledge, this is the largest study to examine this intervention while still retaining the individualisation process central to their design. We have successfully developed and refined a Social Stories training package for use in mainstream school. This training package which consists of a manual and a training day has the potential to help children with ASD overcome some of the social difficulties they experience at school in a non-expensive non-intrusive way, subject to its being shown to be effective and cost-effective in a future trial. Were this to be demonstrated, the intervention could be made widely available to educational and community settings across the country.
